# The Early Diagnosis of Insanity

**Published:** 1896-11-07

**Authors:** F. St. John Bullen

**Affiliations:** formerly Pathologist and Medical Officer, West Riding Asylum, Wakefield.


### THE EARLY DIAGNOSIS OF INSANITY.

By F. St. John Bullen, M.R.C.S., formerly Pathologist and Medical Officer, West Riding Asylum, Wakefield.

The practitioner will occasionally be consulted by or concerning patients in whom abnormal mental symptoms are appearing, and in all instances he will be asked to give an opinion as to whether these imply the advent of insanity or no. This, partly with a view to ending suspense, partly in hope of preventive treatment. It is the object of this brief article to allude to various points which will serve as a guide in forming a diagnosis. This, in truth, cannot be arrived at too early, for at the commencement of insanity neglect or improper treatment may leave unchecked or even aggravate the malady. In many cases it will be found that the patient's symptoms may, with sufficient correctness, be comprised under "neurasthenia"; but as this so-called functional disorder is frequently a prodroma of insanity every care must be exercised to recognise the special potentialities in the individual for developing a morbid psychosis. It is, therefore, imperative to know, on the one hand, the confines of neurasthenia, and, on the other hand, all circumstances which favour the development, or indicate the existence of a definite insanity. To settle the former question demands a thorough acquaintance with the affection, with its underlying principle of "irritable weakness," the sensory and motor anomalies, various phobias, imperative ideas, and idiosyncrasies. It is, then, possible to trace its transition into insanity, recognising false interpretations to the various dysæsthesiæ, morbid emotional states, loss of control, and failure of correct relation to environment.

Whilst heredity is found both in neurasthenia and insanity its influence is not, of course, equally potent in both. In the less grave condition the effects appear in a slightly defective development of the nervous system as a whole, which together, perhaps, with some similar degree of under-development of the vascular system, results in a chronic insufficiency of nervous energy with abnormal sensitiveness and irritability. The gravest form of neurasthenia, and one which in some points transcends its proper limits, is seen in persons more strongly tainted by heredity, the class termed by Magnan "Dégénerés." These unfortunates, who have received physical as well as mental stigmata, are often the victims of harassing imperative ideas and impulses, which arise in minds painfully conscious of the transient disequilibrations. This great class requires entire recognition, giving the clue, as it does, to the origin of apparently motiveless acts, to the various morbid cravings, indicating the mental soil upon which chronic and persistent delusory states engraft themselves, and further, suggesting the gloomy prognosis which must be given in these cases.

Having fully determined the existence of the neurasthenic state, it must be enquired whether this is likely to develop into insanity. The disposal of this question will largely rest upon a knowledge of all the predisposing and exciting causes of insanity. Or, the patient may have already passed the bounds of sanity. This can only be decided by the complete acquaintance of the physician with the patient's antecedents, former habits, changes in environmental reaction, largely also with the observer's ability to grasp even slight departures from a normal mental state, and from their aggregate to obtain suggestive or decisive information. It must be acknowledged that there are but few pathognomonic symptoms by which a threatening attack of insanity may be foretold. Sooner or later a stage will be reached when can be noticed in the patient a change, antagonistic to the former feelings, desires, conduct, habits, and opinions; a change which at the outset is, in whole or in part, perceived by the patient himself. Should this alteration be gradual, it may for a long time be inseparable from the growth of firm convictions in an ordinary sane individual; or it may be regarded as the emphasis of some known peculiarity which seems assuming ascendancy over the thoughts and conduct. Insomnia, troublous dreams, irascibility, inconsistencies of behaviour, perhaps hallucinations, are familiar ominous symptoms, but do not with certainty indicate the onset of insanity. Hence the importance of carefully weighing the chances caused by (1) the patient's original nervous constitution; (2) his period in regard to mental development, maturity, or dissolution; and (3) various episodic conditions. These will, therefore, now be dealt with.

The patient's nervous organisation is estimated by his heredity. Admittedly powerful factor as this is, it appears as a cause in only 23·2 per cent. of English lunatics in the mass (Commissioners' Tables, 1896). Notwithstanding that this is probably a considerable under-statement, it tends to show that heredity must not be over-estimated as a predisponent to insanity. Its influence is more evident in some psychoses than others, and it is therefore important to group these approximately. To ascertain the existence and strength of heredity, the search must be carried not only through the trunk but the branches of the ancestral tree, the alternation of neuroses must be kept in mind together with their varieties. The stronger the heredity, the earlier in life will be its influence felt, the more marked will be the psychic and other stigmata, the more rapid the onset and the less adequate the exciting cause of the insanity. It is also to be expected that at periods of physiological instability, heredity should assert itself strongly; therefore, towards the climax of development, and at partial and general dissolutions (climacteric and senile periods), and at certain periodic and episodic occurrences, such as menstruation and the puerperium, the tendency to the appearance of psychoses will be manifest. The liability to insanity at certain periods of life is, in the main, but another expression of this recurring instability. There is, in addition, what may be termed the period of stress, which confers its distinctive risk apart from the stage of development in the individual. Thus far for the predisposing influence of heredity and age. There are many causes of "excitation," some seeming sufficiently potent without a prepared soil, the majority, doubtless, not so. Nevertheless, they require consideration. It is natural that conditions with which delirium is associated should occasionally form a starting-point for insanity; accordingly, febrile disorders, as pneumonia, acute rheumatism, the eruptive fevers, typhoid and typhus especially, influenza, may all initiate this in an acute or insidious form. The influence of phthisis upon the production of insanity in the individual is not well settled; it more often shows its existence after mental disorder is well established, so that the relationship is uncertain. Unless cardiac disease, in a predisposed person, causes much discomfort and anxiety it is not likely to cause insanity; arterial atheroma, alone or with heart trouble, is a decided precipitant of mental disorder. Other rare causes, such as gout, malaria, &c., can pass with mere mention. Of the principal toxic conditions, as chronic Bright's disease, syphilis, alcohol, there is more or less to be said—less concerning the first, in which a delirious type of insanity may occur towards the end, or melancholia at an earlier stage, when heredity and alcoholic habits are conjoined; more with regard to syphilis, which is important both as a physical and moral factor. Neuro-vascular changes leading to loss of memory, irritability, explosiveness, despondency, may, especially if co-existent with personal disfigurements, produce serious melancholia. In individuals, married or engaged, who realise the cause of their condition, this is to be particularly dreaded. There is, too, weighty evidence in favour of a relationship to general paralysis. Syphilophobia, without evidence of syphilis, belongs to the neurasthenic group, but may develop into insane depression. So well known an influence as that of alcohol needs little mention, but it is important before giving an opinion on the case to ascertain the ancestral history. It is said that two-thirds of drinkers are the sons of drinkers (apart from the neuroses transmitted). The evidence of heredity will help in ascertaining and explaining the signs of degeneracy. Thus, easy excitability or marked depression under the effects of alcohol, fleeting delirious ideas, nightmare, or stupidity after even slight excesses, will give warning that the patient is overstepping the border line of sanity.

Enquiry should always be made as to cranial injury, which has a very distinct though not a wide influence. Whether the subject of this be neurotic or no, symptoms of disturbed mental action, occurring months or years after the head injury, such as vertigo, forgetfulness, irritability, change in disposition and habit, the committal of some "bizarre" act should be regarded with suspicion. There is no certain relation between the gravity of the accident and that of the psychosis.

After coarse organic lesions in the brain, *e.g.*, hæmorrhage, thrombus, tumour, acute meningitis, dementia may forthwith, slowly or quickly, supervene; or emotional perturbations succeed to delirium or coma following apoplexy.

Certain nervous diseases, notably locomotor ataxy, chorea, and paralysis agitans, occasionally have a sequel of insanity; chorea shows this most frequently. That general paralysis may follow on tabes dorsalis is well known. Epilepsy, when severe, proceeds from the commencement hand in hand with mental reduction. Diverse morbid symptoms have been met with in neurotic subjects at the first, or even a subsequent appearance of menstruation. The mental state may vary from mere unreasonable emotionalism to one of active disorder; moral perversion has been especially noted. In similar subjects, uterine disorders have an undoubted influence in the eventual production of insanity. Liability to a morbid psychosis during the latter years of adolescence has been hinted. Although by far the majority of cases arise between the ages of twenty-one and twenty-five, comparatively few may occur after puberty. In both girls and youths hereditarily predisposed, serious apprehension should be caused by such symptoms as uncalled-for depression, absurd sentimentality and self-consciousness, capricious behaviour, moping, indolent habits; or, on the other hand, unruly obstinacy, fitful excitability, lowered morale, and impaired sleep. In over half the cases a narrow-arched palate is found.

Heredity, together with vascular degeneration, exhausting disease, or over-taxation of failing energies, may effect one of the senile psychoses, which are the same in type as those at other periods of life, though modified by brain decrepitude. The prodromata are mainly the well-known ones of ordinary senility, with nocturnal restlessness, hallucinations, morbid apprehensions, &c., added. Women, unless gravely predisposed, very seldom become insane during pregnancy. Altered disposition to husband and children, with insomnia, depression, restlessness, nightmare, occurring after childbirth in a neurotic woman (especially if primapara over thirty, or after a trying labour), or during lactation in a debilitated mother similarly predisposed, are ominous of approaching insanity. Over half the cases of insanity at the climacteric show well-marked heredity. There is, then, a great proneness to mental breakdown, and any aggravation of the common sympathetic and mental disturbances of this era, where there is neurotic history, is serious. Such may show itself in sleeplessness, depression, irritability, suspiciousness, jealousy, lowered morale, hallucinations, and tendency to irrational interpretation of novel sensations.

When a man, preferably between the ages of thirty and forty-five, who has been subjected to mental over-strain for years, who has, with or without this stress, lived fast, in whom alcohol and other excesses, syphilis, or cranial injury have played a part, displays suddenly or slowly a marked alteration in his normal conduct and habits, disposition and temper, and when, perhaps, acts of glaring inconsistency or moral depravity are exhibited unconcernedly, there must always be grave suspicion of general paralysis. The impairment of moral sense shown gradually or by some impulsive, even criminal, act, is, on the whole, the earliest and most symptomatic warning of this disease. Some form of convulsive seizure, transient paresis of speech, limb, or cranial nerve is equally suggestive. Physical symptoms as prodromata are often absent at an early stage, hence articulatory and pupillary impairments may not aid the diagnosis. It is important to remember that a melancholic and hypochondriacal form of general paralysis is not seldom met with, and far from spendthrift, wild, uncalculating conduct in business or pleasures, ideas of impending poverty, ruin, or shattered health, torpor, and indolence may usher in the disease.

In those cases of somnambulism in which the acts committed show evidence of certain persistently recurring delusory ideas and hallucinations, there is less fear of insanity than of the occurrence of dangerous impulses. The temporary abeyance of highest consciousness caused by sleep seems to reveal serious potentialities which, though hidden during waking hours, may under favouring circumstance, such as great emotionalism, exhibit themselves in an explosive manner.

